# Long-Term Drug Screening Identifies PARP Inhibitors as Irinotecan Combination Partners in BRCA1/2-Wild-Type Pancreatic Ductal Adenocarcinoma

**DOI:** 10.3390/ph19101538

**Published:** 2026-09-28

**Authors:** Hyeweon Jang, Hansol Jang, Hye Yeon Yun, Kyung Yong Lee, Hyonchol Jang

**Affiliations:** 1 Division of Cancer Biology, Research Institute, National Cancer Center, Goyang 10408, Republic of Korea; jhw4949@ncc.re.kr (H.J.); 76814@ncc.re.kr (H.J.);; 2 Department of Cancer Biomedical Science, National Cancer Center Graduate School of Cancer Science and Policy, Goyang 10408, Republic of Korea

**Keywords:** pancreatic ductal adenocarcinoma, irinotecan, PARP inhibitor, BRCA1/2-wild-type, drug combination, drug screening, clonogenic assay

## Abstract

**Background/Objectives:** Irinotecan is a component of standard first-line regimens for advanced pancreatic ductal adenocarcinoma (PDAC), yet cells that survive irinotecan treatment may retain the capacity to re-establish growth. Most anticancer drug screens use short-term growth inhibition as an initial criterion for compound selection, potentially excluding compounds with limited acute activity. We asked whether some of these excluded candidates might nevertheless show substantial long-term activity and enhance irinotecan responses in combination. **Methods:** We first screened 3524 compounds for short-term growth inhibition and advanced compounds with limited acute activity to a 10-day colony-growth screen. Long-term hits were narrowed to clinically relevant anticancer agents and then screened with irinotecan. Talazoparib showed the lowest combination-to-single-agent score and was selected for further evaluation. The combination was evaluated across multiple BRCA1/2-wild-type PDAC cell lines and further validated using additional PARP inhibitors and a xenograft model. RNA sequencing and γH2AX analysis were used to examine associated DNA damage-response features. **Results:** Of the 1877 compounds advanced from the initial 3-day screen, 334 (17.8%) met the long-term hit criterion. In short- versus long-term comparisons, irinotecan plus talazoparib produced greater growth suppression and higher Bliss synergy scores in 10-day than in 72 h assays. Enhanced long-term responses were observed across five BRCA1/2-wild-type PDAC cell lines, and similar long-term effects were observed with three additional PARP inhibitors. The combination also showed synergistic antitumor activity in an MIA PaCa-2 subcutaneous xenograft model. Transcriptomic and γH2AX analyses provided findings consistent with replication-stress and DNA-damage-response processes. **Conclusions:** A substantial subset of compounds with limited acute activity showed substantial long-term activity. PARP inhibition enhanced irinotecan responses and emerged as a candidate combination strategy in BRCA1/2-wild-type PDAC.

## 1. Introduction

FOLFIRINOX and NALIRIFOX are established first-line regimens for metastatic pancreatic ductal adenocarcinoma (PDAC), alongside gemcitabine plus nab-paclitaxel [1,2,3]. Both irinotecan-containing regimens can produce tumor regression and prolong survival, yet most patients eventually develop progressive disease [1,2,4]. A central therapeutic problem is that, despite initial reduction in tumor burden, surviving cells may retain the capacity to sustain or re-establish tumor growth after treatment [5,6]. In patient PDAC, residual tumor-cell states enriched after chemotherapy have been associated with poor outcomes following modified FOLFIRINOX [5]. These observations highlight the importance of considering treatment effects beyond the initial response when identifying irinotecan combination partners.

Irinotecan stabilizes covalent topoisomerase I–DNA cleavage complexes, and replication-fork collision with these complexes generates DNA breaks and replication stress [7,8]. The effects of DNA-damaging agents and poly(ADP-ribose) polymerase (PARP) inhibitors can increase markedly with exposure duration [9,10]. Short-term growth assays may therefore underestimate effects that emerge during prolonged treatment, whereas clonogenic assays provide a complementary readout of long-term growth suppression [11]. Most large-scale anticancer screens nevertheless begin with short-duration growth or viability assays [12,13]. Because conventional clonogenic assays are relatively time- and labor-intensive and can be difficult to scale for high-throughput screening [14,15], compounds with limited acute activity may be discarded before their long-term or combination effects are examined. This creates a specific blind spot for compounds with limited short-term activity but substantial effects during prolonged treatment. We therefore inverted the usual selection logic by excluding compounds with strong short-term activity and advancing those with limited acute effects to long-term colony-growth screening.

This strategy identified PARP inhibition as a candidate for further investigation in BRCA1/2-wild-type PDAC. Maintenance olaparib is approved for selected patients with germline BRCA1/2-mutated metastatic PDAC whose disease has not progressed during first-line platinum-based chemotherapy [16]. PARP inhibitors have also been clinically explored in combination with irinotecan-containing chemotherapy in pancreatic cancer [17,18]. However, the therapeutic relevance of PARP inhibition beyond BRCA1/2-mutated PDAC remains incompletely defined [19]. Moreover, previous studies of irinotecan–PARP inhibitor combinations have not directly examined whether combination effects become more apparent during prolonged treatment [17,18,20,21].

We therefore asked whether sequential short- and long-term screening with clinically oriented prioritization could identify an irinotecan combination partner that would not be prioritized by acute single-agent screening and whether prolonged treatment would reveal stronger combination activity across BRCA1/2-wild-type PDAC models and multiple PARP inhibitors. We further evaluated the combination in vivo and examined DNA damage-response features associated with the treatment response.

## 2. Results

### 2.1. Sequential Short- and Long-Term Screening Identifies Talazoparib as an Irinotecan Combination Candidate

A total of 3524 compounds were initially screened in MIA PaCa-2 cells at 5 μM using a 3-day growth assay. This initial screen was designed to exclude compounds with substantial acute single-agent activity. Percentage growth was calculated relative to the time-zero and vehicle-treated controls using the NCI method, and 1877 compounds with percentage growth of at least 80% were selected for subsequent long-term screening (Figure 1A,B).

The 1877 selected compounds were then tested at the lower concentration of 1 μM for 10 days in a long-term colony-growth screen. Colony growth was assessed semiquantitatively by phase-contrast microscopy relative to vehicle-treated controls. Among the 1877 compounds, 334 compounds (17.8%) were classified into the two strongest-response categories, corresponding to an estimated ≤30% of vehicle-control growth. These compounds were identified as screening hits and subjected to further clinical prioritization (Figure 1A,C).

To focus the subsequent combination screen on clinically relevant agents, the 334 long-term hits were prioritized based on U.S. Food and Drug Administration (FDA) cancer approval status and clinical relevance. FDA-approved anticancer agents were retained, whereas conventional cytotoxic chemotherapies were excluded. When multiple agents shared the same primary molecular target, a single representative agent was selected to avoid redundancy, yielding 18 compounds for subsequent irinotecan combination screening (Figure 1A) [22,23].

To reduce the contribution of single-agent activity during combination screening, the concentration of the 18 selected compounds was reduced to 0.1 μM. The compounds were tested for 10 days, either alone or in combination with 0.5 μM irinotecan (Figure 1A,D). Compounds were ranked by the combination-to-single-agent (C/S) score, calculated as combination relative growth divided by single-agent relative growth [24]. Talazoparib showed the lowest C/S score and was selected for further validation. The established clinical relevance of PARP inhibition in PDAC provided additional rationale for its selection [16].

### 2.2. Long-Term Dose-Matrix Analysis Shows Synergy Between Irinotecan and Talazoparib in MIA PaCa-2 Cells

To further validate the combination activity observed in the single-concentration screen, talazoparib was evaluated across a range of concentrations in a 10-day dose–response matrix in MIA PaCa-2 cells. Whereas the preceding screening steps were performed in 96-well plates, this validation experiment was conducted in 12-well plates to allow more quantitative assessment of long-term clonogenic growth and was performed in three independent experiments. Irinotecan was applied at 0, 0.5, or 1 μM and talazoparib at 0–1 μM (Figure 2A).

Long-term cell growth was quantified by crystal violet staining, and the resulting values were normalized to the vehicle-treated control (Figure 2B). Combination treatment resulted in greater suppression of long-term cell growth than either single agent at multiple dose combinations, with strong positive Bliss synergy scores particularly when 0.5 μM irinotecan was combined with 1–100 nM talazoparib (Figure 2B,C). At 0.5 μM irinotecan and 1 nM talazoparib, each agent produced little growth suppression when used alone, whereas the combination reduced long-term cell growth to approximately 13% of the vehicle-treated control (Figure 2D). This finding shows that talazoparib strongly enhanced the long-term effect of irinotecan at concentrations with minimal single-agent activity.

### 2.3. Long-Term Clonogenic Assays Reveal More Pronounced Irinotecan–Talazoparib Synergy in PANC-1 and AsPC-1 Cells

Following the long-term validation in MIA PaCa-2 cells, we next examined whether the irinotecan–talazoparib interaction was more evident under long-term than short-term assay conditions. PANC-1 and AsPC-1 cells were evaluated using 72 h SRB assays and 10-day clonogenic assays. For direct comparison, overlapping concentration ranges from the short- and long-term assays are shown in Figure 3, with additional concentration-response data provided in Figure S1. In PANC-1 cells, short-term treatment produced modest positive Bliss synergy scores at selected dose combinations, whereas the 10-day assay showed marked suppression of colony formation and substantially higher positive Bliss synergy scores, particularly at low talazoparib concentrations (Figure 3A,B and Figure S1A,E). At the concentration pair selected for direct comparison between the two assay durations, Bliss synergy scores were higher in the 10-day assay than in the 72 h assay (Figure 3C). A similar pattern was observed in AsPC-1 cells. Positive Bliss synergy scores were detected at selected dose combinations in the short-term assay, whereas prolonged treatment resulted in marked suppression of colony formation and stronger positive Bliss synergy scores across several low-dose combinations (Figure 3D,E and Figure S1B,F). Bliss synergy scores at the matched concentration pair were likewise higher in the 10-day assay than in the 72 h assay (Figure 3F). Together, these results demonstrate that irinotecan plus talazoparib produced stronger effects and higher Bliss synergy scores in 10-day clonogenic assays than in 72 h SRB assays in both PANC-1 and AsPC-1 cells.

### 2.4. Long-Term Responses to Irinotecan Plus Talazoparib Extend to Additional BRCA1/2-Wild-Type PDAC Cell Lines

To determine whether the enhanced long-term response to irinotecan plus talazoparib extended to additional PDAC models, two additional BRCA1/2-wild-type cell lines, BxPC-3 and HPAC, were evaluated using 10-day dose–response matrices. In both cell lines, treatment with irinotecan plus talazoparib suppressed long-term colony formation across multiple concentration pairs (Figure 4A,C and Figure S1G,H). Bliss analysis yielded positive synergy scores at selected concentration pairs in both BxPC-3 and HPAC cells (Figure 4B,D). Together with the preceding results from MIA PaCa-2 (Figure 2C), PANC-1 (Figure 3B), and AsPC-1 (Figure 3E) cells, these findings show that enhanced long-term responses to irinotecan plus talazoparib, accompanied by positive Bliss synergy scores, were observed across all five BRCA1/2-wild-type PDAC cell lines examined.

### 2.5. Multiple PARP Inhibitors Show Similar Long-Term Effects with Irinotecan in MIA PaCa-2 and PANC-1 Cells

To determine whether the enhanced long-term effect observed with talazoparib was shared by other PARP inhibitors, three additional PARP inhibitors—olaparib, niraparib, and rucaparib—were evaluated with irinotecan in MIA PaCa-2 and PANC-1 cells using 10-day clonogenic assays. In MIA PaCa-2 cells, irinotecan paired with each PARP inhibitor suppressed long-term colony formation across multiple concentration pairs, consistent with the corresponding relative long-term growth measurements (Figure 5A,C,E and Figure S2A,C,E.). Bliss analysis showed positive synergy scores at multiple concentration pairs for olaparib, niraparib, and rucaparib (Figure 5A,C,E). Similar patterns of long-term growth suppression and positive Bliss synergy scores were observed in PANC-1 cells (Figure 5B,D,F and Figure S2B,D,F). For each PARP inhibitor, Bliss synergy scores from the three independent experiments are shown at a representative concentration pair (Figure 5G,H). Together, these findings demonstrate that enhanced long-term responses to irinotecan were reproducibly observed with multiple PARP inhibitors in both PDAC cell lines.

### 2.6. Talazoparib Enhances the Antitumor Activity of Irinotecan in an MIA PaCa-2 Subcutaneous Xenograft Model

To evaluate antitumor activity in vivo, MIA PaCa-2 subcutaneous xenografts were treated with vehicle, irinotecan at 20 mg/kg, talazoparib at 0.15 mg/kg, or the combination (*n* = 5 mice per group; Figure 6A). Tumor growth was attenuated by each single agent but continued to increase during treatment, whereas the combination markedly restricted tumor expansion over the treatment period. invivoSyn analysis using the Bliss independence model demonstrated significant in vivo synergy, with a combination index of 0.441 (95% bias-corrected and accelerated (BCa) confidence interval, 0.123–0.871; *p* = 0.008) and a synergy score of 18.752 (95% BCa confidence interval, 3.738–41.795; *p* = 0.008; Figure 6B). Endpoint tumor weights were lower in the combination group than in either single-agent group (Figure 6C), consistent with the smaller excised tumors observed at endpoint (Figure 6D). No marked body weight loss was observed during treatment (Figure 6E). Together, these findings demonstrate a significant synergistic antitumor effect of irinotecan and talazoparib in the MIA PaCa-2 subcutaneous xenograft model.

### 2.7. Irinotecan-Conditioned Cells Show Enrichment of Replication-Stress and DNA-Repair Gene Sets

To provide biological context for the enhanced response to irinotecan plus talazoparib, we next examined transcriptional changes associated with repeated irinotecan exposure. Because irinotecan-containing regimens used in PDAC administer irinotecan intermittently rather than continuously [1,2], we used repeated treatment and recovery periods to model this alternating exposure pattern. This design was used to examine cellular responses associated with repeated irinotecan exposure and recovery, rather than to directly compare therapeutic dosing schedules. We generated two irinotecan-conditioned populations representing a drug-free recovery state and a state of renewed irinotecan exposure. MIA PaCa-2 cells were subjected to two irinotecan-conditioning schedules (Figure 7A). In Schedule A, cells underwent two cycles of irinotecan treatment and recovery and were collected after the final recovery period (recovered cells), whereas in Schedule B, cells underwent two treatment–recovery cycles followed by a final irinotecan re-exposure and were collected immediately thereafter (re-exposed cells). RNA sequencing (RNA-seq) was performed on these irinotecan-conditioned populations and parental cells. Both recovered and re-exposed cells showed enrichment of gene sets related to replication stress, DNA synthesis, cell-cycle checkpoints, and DNA repair (Figure 7B).

Gene set enrichment analysis (GSEA) further showed enrichment of pathways related to the ataxia telangiectasia and Rad3-related (ATR) replication-stress response, AP-site/base-excision repair, and homology-directed repair in both experimental sets (Figure 7C). Genes contributing to the core enrichment of these pathways showed similar GSEA rank metric patterns across the two schedules (Figure 7D). Similar pathway enrichment was observed in both Recovered and Re-exposed cells, indicating that these transcriptional features were detectable after drug-free recovery as well as following irinotecan re-exposure. These observations were considered exploratory because RNA sequencing was performed on one sample per condition.

To assess DNA damage in vivo, xenograft tissues from vehicle, irinotecan, talazoparib, and combination-treated mice were examined by hematoxylin and eosin (H&E) staining and phosphorylated histone H2AX at Ser139 (γH2AX) immunohistochemistry (IHC) (Figure 7E). Quantitative analysis showed that γH2AX staining was highest in the irinotecan–talazoparib combination group (Figure 7F), consistent with increased DNA-damage accumulation under combination treatment.

## 3. Discussion

This study used a sequential short- and long-term screening strategy designed to retain compounds with limited acute single-agent activity and identify irinotecan combination partners whose effects may become more apparent during prolonged treatment. Following long-term colony-growth screening and clinical prioritization, talazoparib showed the lowest C/S score in the fixed-concentration irinotecan combination screen and was selected for further investigation, with its clinical relevance in PDAC providing additional rationale for prioritization. Enhanced responses to irinotecan plus talazoparib were subsequently observed across five BRCA1/2-wild-type PDAC cell lines, and similar long-term effects were observed when irinotecan was paired with multiple PARP inhibitors. The combination further showed synergistic antitumor activity in an MIA PaCa-2 subcutaneous xenograft model.

These findings suggest that compounds with limited acute single-agent activity may include a substantial number of candidates with meaningful long-term or combination activity. In our study, 334 of 1877 compounds (17.8%) showing limited activity in the initial 3-day screen met the long-term hit criterion, even at a lower concentration (1 μM versus 5 μM). Similar differences between short- and long-term drug responses have been reported in previous studies [9,25,26,27]. Short-term growth or viability assays remain practical for large-scale screening and continue to be widely used in contemporary combination screens [12,13]. The present findings therefore suggest that weak acute single-agent activity alone should not be used to dismiss a compound when its potential value lies in prolonged treatment or combination therapy. Such compounds may merit re-evaluation as combination partners or when long-term growth suppression is a therapeutically relevant endpoint. This possibility was illustrated by irinotecan plus talazoparib in PANC-1 and AsPC-1 cells, where modest effects in 72 h assays contrasted with substantially greater growth suppression and higher Bliss synergy scores in 10-day clonogenic assays.

The enhanced response to irinotecan plus PARP inhibition also raises the question of whether PARP inhibitors may have value as combination partners beyond tumors that are sensitive to PARP inhibition alone. In PDAC, the clinical benefit of PARP inhibition has been most clearly established in tumors with germline BRCA1/2 mutations [16,19]. However, sensitization to topoisomerase I inhibitors by PARP inhibition is not necessarily restricted to settings in which PARP inhibitors show strong single-agent activity, and preclinical studies have demonstrated enhanced responses to topoisomerase I inhibition at PARP inhibitor concentrations with limited activity on their own [20]. Consistent with this concept, enhanced long-term responses to irinotecan plus talazoparib were observed across all five BRCA1/2-wild-type PDAC cell lines examined here, and similar effects were obtained with olaparib, niraparib, and rucaparib. These findings indicate that the enhanced long-term combination effect was not limited to talazoparib among the PARP inhibitors tested. However, the magnitude of the combination response may vary depending on the PARP inhibitor, dose, and treatment schedule. Together, these observations suggest that PARP inhibitors may have broader potential as irinotecan combination partners in PDAC than would be predicted from their single-agent activity alone.

Previous clinical experience nevertheless shows that translating this interaction is challenging. In the randomized SWOG S1513 study, adding veliparib to modified FOLFIRI did not improve clinical outcome in an unselected metastatic PDAC population [18], and earlier irinotecan–PARP inhibitor studies encountered substantial treatment-related toxicity [17,21]. These findings make dose and schedule important considerations for this strategy. In the present study, irinotecan was administered at 20 mg/kg once weekly, whereas preclinical PDAC studies have commonly used approximately 25–50 mg/kg/week or higher dose intensities in irinotecan-containing regimens [28,29]. Talazoparib was administered at 0.15 mg/kg five times weekly, within the range of doses used in previous xenograft studies, including doses of approximately 0.1–0.33 mg/kg [30,31]. More recent studies have continued to investigate topoisomerase I–PARP inhibitor combinations using intermittent PARP inhibition, modified scheduling, or altered drug delivery [32,33]. Taken together, these findings support continued evaluation of irinotecan–PARP inhibitor combinations in PDAC, including systematic assessment of dose, treatment sequence, and schedule to further optimize combination activity.

The transcriptional changes observed after repeated irinotecan exposure provide biological context for the enhanced response to irinotecan plus PARP inhibition during prolonged treatment. Topoisomerase I inhibitors generate TOP1–DNA cleavage complexes that interfere with replication-fork progression and activate replication-stress responses involving ATR-dependent checkpoint signaling and multiple DNA-repair pathways [7,8,34]. Recent work in PDAC has also shown that base-excision repair contributes to the control of transcription–replication conflicts and that disruption of this pathway increases DNA damage and ATR–CHK1 signaling [35]. In the present study, pathways related to replication stress, cell-cycle checkpoints, and DNA repair were enriched both after irinotecan re-exposure and after a drug-free recovery period. These findings indicate that transcriptional features related to these processes were detectable under both experimental conditions. However, because the transcriptomic analysis was based on one sample per condition, these observations should be regarded as exploratory and cannot establish persistence of a transcriptional state or a causal role for these pathways in the enhanced response to PARP inhibition. PARP1 participates in the repair of TOP1-induced DNA lesions, and PARP inhibition has been reported to increase DNA damage induced by TOP1 inhibitors [20,36]. Together with the increased γH2AX staining observed in combination-treated xenografts, the transcriptomic findings provide biological context consistent with involvement of replication-stress and DNA-damage-response processes.

Several limitations should be considered when interpreting these findings. The transcriptomic analysis was exploratory and was based on one RNA-seq sample per condition. Biological variability could therefore not be estimated, and these data cannot establish reproducible differential expression or identify causal mechanistic drivers. The GSEA findings should instead be considered descriptive pathway-level observations that provide biological context for the treatment response. Because the short- and long-term responses were evaluated using different assay formats as well as different treatment durations, the magnitude of the difference between the two assays should not be interpreted as a direct measure of the effect of prolonged drug exposure alone. Finally, although the in vivo combination activity was demonstrated in the MIA PaCa-2 subcutaneous xenograft model, evaluation in additional PDAC models, including orthotopic and patient-derived xenografts, would further strengthen the translational relevance of these findings.

## 4. Materials and Methods

### 4.1. Cell Lines and Culture Conditions

MIA PaCa-2, PANC-1 and AsPC-1 cells were obtained from the Korean Cell Line Bank (Seoul, Republic of Korea); BxPC-3 and HPAC cells were obtained from the American Type Culture Collection (Manassas, VA, USA). MIA PaCa-2 and PANC-1 cells were maintained in Dulbecco’s modified Eagle medium, and AsPC-1, BxPC-3 and HPAC cells were maintained in RPMI-1640 medium, supplemented with 10% fetal bovine serum and 1% penicillin–streptomycin. Cells were cultured at 37 °C in 5% CO_2_. Cell line identity was confirmed by short tandem repeat profiling, and cultures were routinely tested for mycoplasma contamination at the Genomics Core Facility, National Cancer Center (Goyang, Republic of Korea). MIA PaCa-2, PANC-1, AsPC-1, BxPC-3 and HPAC cells have been reported to be wild type for BRCA1 and BRCA2 [37].

### 4.2. Compounds and Reagents

A custom-assembled compound library comprising 3524 compounds was obtained from MedChemExpress (Monmouth Junction, NJ, USA). The screening collection was customized from compounds included in the MCE Natural Product Library and Clinical Compound Library. Compounds were supplied as 10 mM solutions in dimethyl sulfoxide (DMSO) in 96-well source plates and were stored at −80 °C until use. The complete list of compounds included in the screening collection is provided in Table S1. Irinotecan was purchased from LC Laboratories (Woburn, MA, USA; Cat. No. I-4122). Talazoparib (HY-16106), olaparib (HY-10162), niraparib (HY-10619) and rucaparib (HY-10617A) were purchased from MedChemExpress. Compounds were prepared in DMSO. Vehicle concentrations were matched across treatment conditions.

### 4.3. Short-Term Single-Agent Library Screening

MIA PaCa-2 cells were seeded in 96-well plates at 5000 cells per well and allowed to attach for 24 h. A separate time-zero plate was fixed at the start of drug treatment. Cells were treated with individual compounds at 5 μM for 72 h, and cell growth was measured using the SRB assay (Sigma-Aldrich, St. Louis, MO, USA; Cat. No. S9012) as previously described [38]. Percentage growth was calculated from the time-zero, vehicle-control, and compound-treated absorbance values according to the NCI method [39].

### 4.4. Long-Term Colony-Growth Library Screening

MIA PaCa-2 cells were seeded in 96-well plates at a density of 300 cells per well and allowed to attach for 24 h. The 1877 compounds selected from the short-term screen were individually applied at a final concentration of 1 μM and maintained for 10 days. At the end of treatment, long-term colony growth was assessed semiquantitatively by phase-contrast microscopy without fixation or staining, based on residual adherent cell density and colony growth relative to vehicle-treated wells. Responses were categorized as Group 0 (≤10%), Group 1 (>10–30%), Group 2 (>30–80%), or Group 3 (>80–100%) of the vehicle-treated control. A residual growth threshold of ≤30% was used to define long-term screening hits, balancing the selection of compounds with marked long-term growth suppression with the number of candidates advanced to subsequent prioritization.

### 4.5. Clinical Prioritization of Long-Term Screening Hits

Compounds identified in the long-term screen were further prioritized according to their clinical use. FDA approval status and approved oncology indications were verified using the U.S. Food and Drug Administration Drugs@FDA database [22], and drug classes and mechanisms of action were cross-referenced using the National Cancer Institute Drug Dictionary [23]. FDA-approved anticancer agents were retained, whereas conventional cytotoxic chemotherapies were excluded. When multiple agents shared the same primary molecular target, a single representative agent was selected to avoid redundancy.

### 4.6. Fixed-Concentration Irinotecan Combination Screening

MIA PaCa-2 cells were seeded in 96-well plates at a density of 100 cells per well and allowed to attach for 24 h. Eighteen clinically prioritized compounds were tested at 0.1 μM for 10 days, either alone or in combination with 0.5 μM irinotecan. Irinotecan alone was included as a reference condition. At the end of treatment, cell growth was assessed using the SRB assay, and relative cell growth (%) was calculated by normalization to the vehicle-treated control. The combination-to-single-agent (C/S) score was calculated by dividing combination relative growth by the corresponding single-agent relative growth, and compounds were ranked according to C/S score [24]. The screening was performed as a single experiment with three replicate wells per condition.

### 4.7. Long-Term Clonogenic Assays

Cells were seeded at 500 cells per well in 12-well plates and treated with the indicated compounds for 10 days. Experiments were performed independently three times, with three replicate wells per condition in each experiment. Colonies were fixed and stained in phosphate-buffered saline (PBS) containing 0.02% crystal violet, 1% methanol, and 1% formaldehyde, washed with PBS, and air-dried. Retained crystal violet was solubilized with 1% sodium dodecyl sulfate, and absorbance was measured at 580 nm. Relative clonogenic growth was normalized to the corresponding vehicle-treated control, which was set at 100%. Four-condition assays comparing vehicle, irinotecan, talazoparib, and combination treatment were analyzed using repeated-measures one-way ANOVA with Geisser–Greenhouse correction followed by Šídák’s multiple-comparisons test.

### 4.8. Short-Term SRB Growth Assays

For short-term combination assays, cells were seeded at 5000 cells per well in 96-well plates and treated with the indicated compounds for 72 h. Cell growth was measured using the SRB assay as described above. Endpoint SRB values were normalized to the corresponding vehicle-treated control without time-zero correction and used for Bliss synergy analysis. Experiments were performed independently three times, with three replicate wells per condition in each experiment.

### 4.9. Bliss Synergy Analysis

Pharmacological interactions were quantified using Combenefit software (version 2.021) based on the Bliss independence model [40]. Replicate wells were averaged within each independent experiment, and the resulting normalized response matrices from three independent experiments were analyzed together. Bliss synergy scores were calculated for each concentration pair and are presented as mean ± SD from the three independent experiments. Statistical significance of Bliss synergy scores was calculated by Combenefit using an element-wise one-sample *t*-test against zero. For summary visualization, a representative concentration pair showing positive combination activity without marked single-agent growth suppression was selected for each PARP inhibitor–cell line combination, and scores from the three independent experiments at that concentration pair were plotted. For comparison of the 72 h and 10-day assays, the concentration pair with the highest average Bliss synergy score across both assays was selected. Bliss synergy scores from the three independent experiments were compared at the same concentration pair.

### 4.10. Xenograft Model and Drug Treatment

Five-week-old female BALB/c nude mice were obtained from Orient Bio (Seongnam-si, Gyeonggi-do, Republic of Korea) and maintained under specific pathogen-free conditions. MIA PaCa-2 cells (5 × 10^6^ cells per mouse) were suspended in 100 μL of a 1:1 mixture of PBS and Matrigel (Corning, Cat. No. 354230) and injected subcutaneously into the right flank of each mouse.

When tumor volumes reached approximately 100–200 mm^3^, mice with comparable baseline tumor volumes were selected for treatment and randomly assigned to four groups using a random-number function in Microsoft Excel (*n* = 5 per group; 20 mice in total): vehicle control, irinotecan, talazoparib, and irinotecan plus talazoparib. The individual mouse was considered the experimental unit. The group size (*n* = 5) was predefined in the approved animal study protocol based on prior experience with this xenograft model.

Irinotecan was prepared in 5% DMSO and 95% PBS and administered intraperitoneally at 20 mg/kg in a total volume of 100 μL once weekly. Talazoparib was prepared in 1% DMSO and 99% PBS and administered by oral gavage at 0.15 mg/kg in a total volume of 100 μL five times per week. Mice in the combination group received both agents according to the same dosing schedules. The corresponding vehicle formulations were administered using the same routes, volumes, and schedules as their respective drug treatments. Treatment was continued for 6 weeks.

Tumor size and body weight were measured weekly, and mice were monitored five times per week for signs of distress or adverse events. Tumor volume was calculated as (length × width^2^)/2. All mice were euthanized at the experimental endpoint as previously described [41], and tumors were harvested for tumor weight measurement and subsequent analyses. Investigators were not blinded to treatment allocation during tumor-volume measurements or data analysis.

Tumor volumes at the experimental endpoint were analyzed using Welch’s one-way ANOVA followed by Dunnett’s T3 multiple-comparisons test. Endpoint tumor weights were compared between the combination group and each single-agent group using Welch’s unpaired *t*-tests with Holm–Šídák correction for the two prespecified comparisons. Welch’s tests were used to accommodate potential differences in variance among groups. Adjusted *p* values < 0.05 were considered statistically significant.

### 4.11. In Vivo Synergy Analysis

In vivo drug synergy was evaluated from longitudinal tumor-volume data using the invivoSyn package (version 0.0.0.9000) in R (version 4.5.3), as described by Mao and Guo [42]. Tumor-volume measurements from the vehicle, irinotecan, talazoparib, and irinotecan–talazoparib combination groups were analyzed using the eGR-based approach implemented in invivoSyn. The combination index (CI) and synergy score (SS) were calculated under the Bliss independence model, with eGR estimated from longitudinal tumor-volume measurements over the study period, while the 21-day reference time specified in the invivoSyn framework served solely as a standardization parameter for calculating CI and SS, rather than as an experimentally selected tumor-measurement time point. Statistical uncertainty and significance were assessed by treatment-stratified bootstrap resampling with 1000 iterations. Ninety-five percent confidence intervals were estimated using the bias-corrected and accelerated (BCa) method, and *p* values were obtained from the invivoSyn synergy analysis. CI values <1 and SS values >0 indicate synergy, whereas CI = 1 and SS = 0 represent the null interaction reference.

### 4.12. Generation of Irinotecan-Conditioned Cell Populations

MIA PaCa-2 cells were subjected to two irinotecan-conditioning schedules. In Schedule A, cells were treated with 0.5 μM irinotecan for 4 days, allowed to recover for 10 days, treated again with irinotecan for 4 days, and then allowed to recover for an additional 6 days before collection (Recovered). In Schedule B, cells were treated with 0.5 μM irinotecan for 4 days, allowed to recover for 10 days, treated again with irinotecan for 4 days, allowed to recover for another 10 days, and then re-exposed to irinotecan for 4 days and collected immediately thereafter (Re-exposed). A single untreated parental sample was used as the common reference for both the Recovered and Re-exposed comparisons; separate time-matched parental controls were not included for the two conditioning schedules.

### 4.13. RNA Sequencing and Data Processing

Total RNA extraction, library preparation, RNA sequencing, and initial bioinformatics processing were performed as previously described [43], with minor modifications. Cell pellets were lysed in TRIzol, and total RNA was purified using the RNeasy Micro Kit (Qiagen, Hilden, Germany; Cat. No. 74004). Stranded mRNA libraries were prepared using the TruSeq Stranded mRNA Library Prep Kit (Illumina, Inc., San Diego, CA, USA) and sequenced by Macrogen Inc. (Seoul, Republic of Korea) on an Illumina platform (Illumina, Inc., San Diego, CA, USA) using paired-end 101 bp reads, generating approximately 67–77 million raw reads per sample. One sample was sequenced for each condition: untreated parental, Recovered, and Re-exposed cells. Processed reads were aligned to the human reference genome GRCh38 using HISAT2 v2.1.0 with gene annotations based on NCBI release 109, and gene expression was quantified using StringTie v2.1.3b and reported as transcripts per million (TPM). Genes with TPM ≤ 1 in all three samples were excluded from downstream analysis.

Gene set enrichment analysis was performed using GSEA version 4.1.0 [44] with Reactome gene sets from the C2 curated collection of Molecular Signatures Database (MSigDB) [45,46]. For GSEAPreranked analysis, genes were ranked according to the difference in log_2_(TPM + 1) between each irinotecan-conditioned population (Recovered or Re-exposed) and parental cells. Enrichment was assessed using 1000 gene-set permutations, and normalized enrichment scores (NES) and FDR q-values were reported. Genes shown in the heatmap were selected from the leading-edge subsets of the indicated Reactome pathways. Genes shared between the Recovered-versus-parental and Re-exposed-versus-parental comparisons were ranked by the mean GSEA rank metric across the two comparisons, and the highest-ranking genes from each pathway were displayed. Because only one sample was sequenced per condition, biological variance could not be estimated and formal differential-expression testing was not performed. GSEAPreranked analysis was therefore used for exploratory pathway-level assessment. The reported NES and FDR q-values reflect enrichment relative to the ranked gene list and should not be interpreted as biological-replicate-based statistical evidence of differential pathway activity. Accordingly, the GSEA results were interpreted descriptively and as hypothesis-generating observations.

### 4.14. Histology, Immunohistochemistry and Image Analysis

Xenograft tumor tissues were fixed in 10% neutral-buffered formalin for 24 h, embedded in paraffin, and sectioned at a thickness of 4 μm. H&E staining was performed using a Leica ST5010 automated slide stainer (Leica Biosystems, Wetzlar, Germany) with Hematoxylin H (catalog no. HEMH-OT-1L) and eosin (catalog no. 3610MIRA01) according to a routine automated staining protocol.

IHC was performed using a Leica BOND RX automated staining system (Leica Biosystems). Following deparaffinization and heat-induced epitope retrieval using a citrate-based buffer at pH 6.0, tissue sections were incubated with an anti-γH2AX antibody (Cell Signaling Technology, Danvers, MA, USA; catalog no. 9718S) at a dilution of 1:1000. Antibody binding was visualized using the BOND Polymer Refine Detection kit (catalog no. DS9800) with 3,3′-diaminobenzidine (DAB) as the chromogen, according to the manufacturer’s instructions. H&E and IHC slides were digitally scanned using a Vectra Polaris automated quantitative pathology imaging system (Akoya Biosciences, Marlborough, MA, USA).

γH2AX IHC was quantified using inForm image analysis software (v3.1.0, Akoya Biosciences). DAB intensity was measured within analyzed tumor regions, and the mean intensity for each tumor was used as the biological unit for group-level analysis (*n* = 3 tumors per treatment group). The mean DAB intensity for each tumor was normalized to the mean value of the vehicle-treated group, which was set to 100%, and expressed as relative γH2AX DAB intensity (% of vehicle). Group differences in relative γH2AX DAB intensity were analyzed using one-way ANOVA followed by Tukey’s multiple-comparisons test.

### 4.15. Statistical Analysis

Unless otherwise stated, data are presented as mean ± SD. For in vitro validation experiments, replicate wells were averaged within each independent experiment, and the independent experiment was used as the unit of statistical analysis. Statistical analyses were performed using GraphPad Prism version 10.6.1 (GraphPad Software, Boston, MA, USA), unless otherwise specified. All Prism-based statistical tests were two-sided, and *p* < 0.05 was considered statistically significant.

### 4.16. Use of Generative Artificial Intelligence

During manuscript preparation, ChatGPT (OpenAI; GPT-5 series) was used for language editing and to assist in preparing the schematic overview shown in Figure 1A based on the experimental workflow specified by the authors. It was not used to generate or analyze experimental data. All AI-assisted output was critically reviewed and edited by the authors.

## 5. Conclusions

This study shows that compounds with limited acute single-agent activity may nevertheless have substantial long-term or combination activity and may therefore be overlooked when candidate prioritization relies primarily on short-term drug responses. Sequential short- and long-term screening identified PARP inhibition as an irinotecan combination strategy, and enhanced long-term responses were observed across multiple BRCA1/2-wild-type PDAC cell lines and with several PARP inhibitors. Irinotecan plus talazoparib also showed synergistic antitumor activity in an MIA PaCa-2 subcutaneous xenograft model, while the transcriptional and γH2AX findings provided biological context consistent with replication-stress and DNA-damage-response processes. Together, these findings support further evaluation of PARP inhibitors as irinotecan combination partners in PDAC, including BRCA1/2-wild-type settings in which PARP inhibitors have limited single-agent activity.

## Figures and Tables

**Figure 1:**
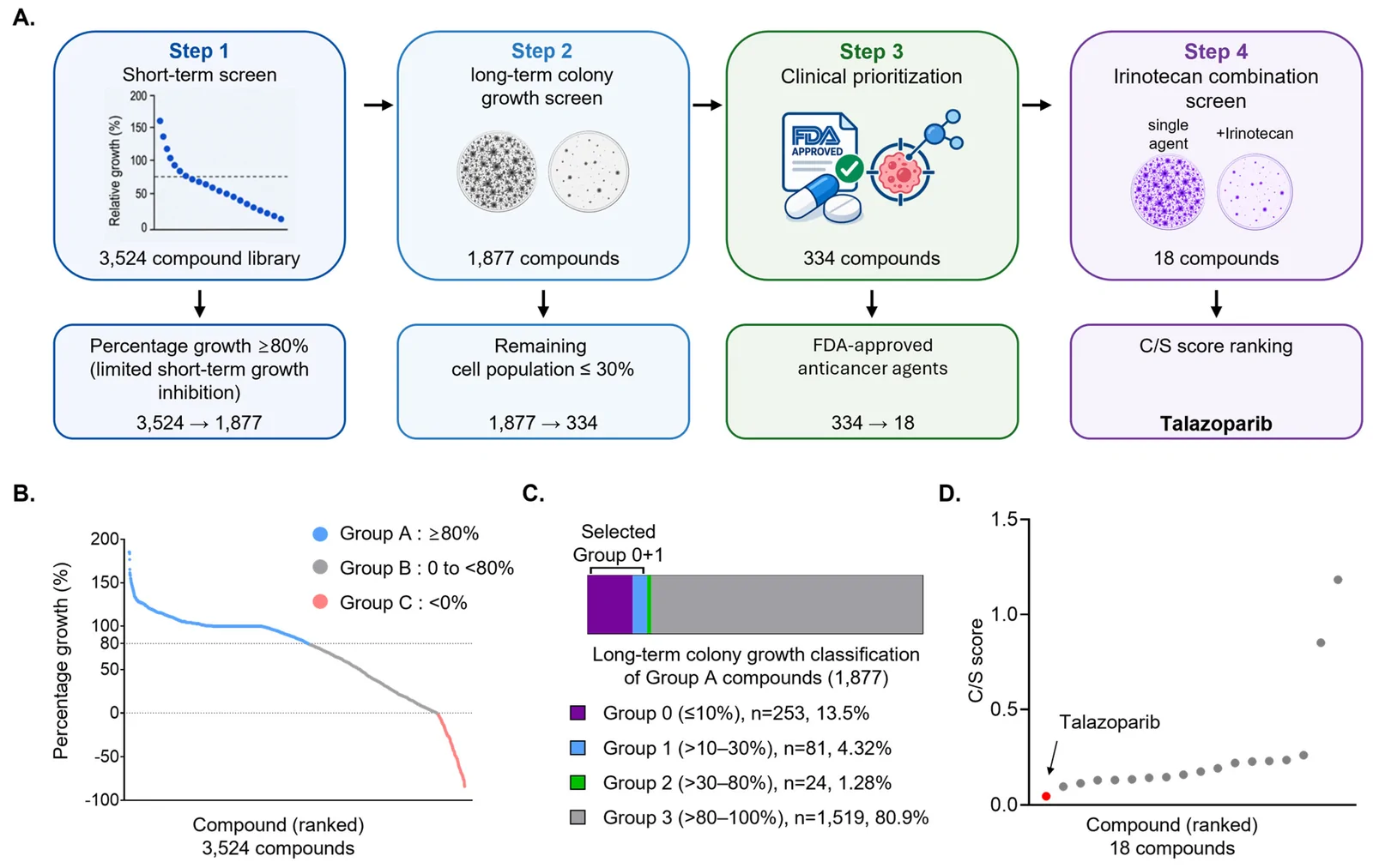
Sequential short- and long-term screening identifies talazoparib as an irinotecan combination candidate in PDAC cells. (**A**) Schematic overview of the sequential screening workflow, comprising short-term screening of a 3524-compound library, long-term colony-growth screening, clinical prioritization, and fixed-concentration combination screening with irinotecan. (**B**) Ranked short-term response profile of the 3524-compound library in MIA PaCa-2 cells. Cells were treated with each compound at 5 μM for 72 h, and cell growth was measured using the SRB assay. Percentage growth was calculated from time-zero, vehicle-control, and compound-treated values according to the NCI method. Compounds were classified as Group A (≥80%), Group B (0 to <80%), or Group C (<0%) based on the resulting relative net-growth values. (**C**) Classification of long-term colony-growth responses among the 1877 compounds in Group A from the short-term screen. MIA PaCa-2 cells were treated with each compound at 1 μM for 10 days, and long-term colony growth was assessed semiquantitatively by phase-contrast microscopy relative to vehicle-treated controls. Responses were categorized as Group 0 (≤10%), Group 1 (>10–30%), Group 2 (>30–80%), or Group 3 (>80–100%) of the vehicle-treated control. Compounds in Groups 0 and 1, corresponding to an estimated 30% or less of vehicle-control growth, were advanced to clinical prioritization. (**D**) The 18 compounds selected through the preceding screening and prioritization steps were subjected to fixed-concentration irinotecan combination screening. MIA PaCa-2 cells were treated for 10 days with each candidate drug (0.1 μM) alone or in combination with irinotecan (0.5 μM). Endpoint cell growth was measured by SRB assay and expressed relative to the vehicle-treated control. The 18 compounds were ranked by the combination-to-single-agent (C/S) score, defined as combination relative growth divided by corresponding single-agent relative growth; talazoparib had the lowest score and was selected for further validation.

**Figure 2:**
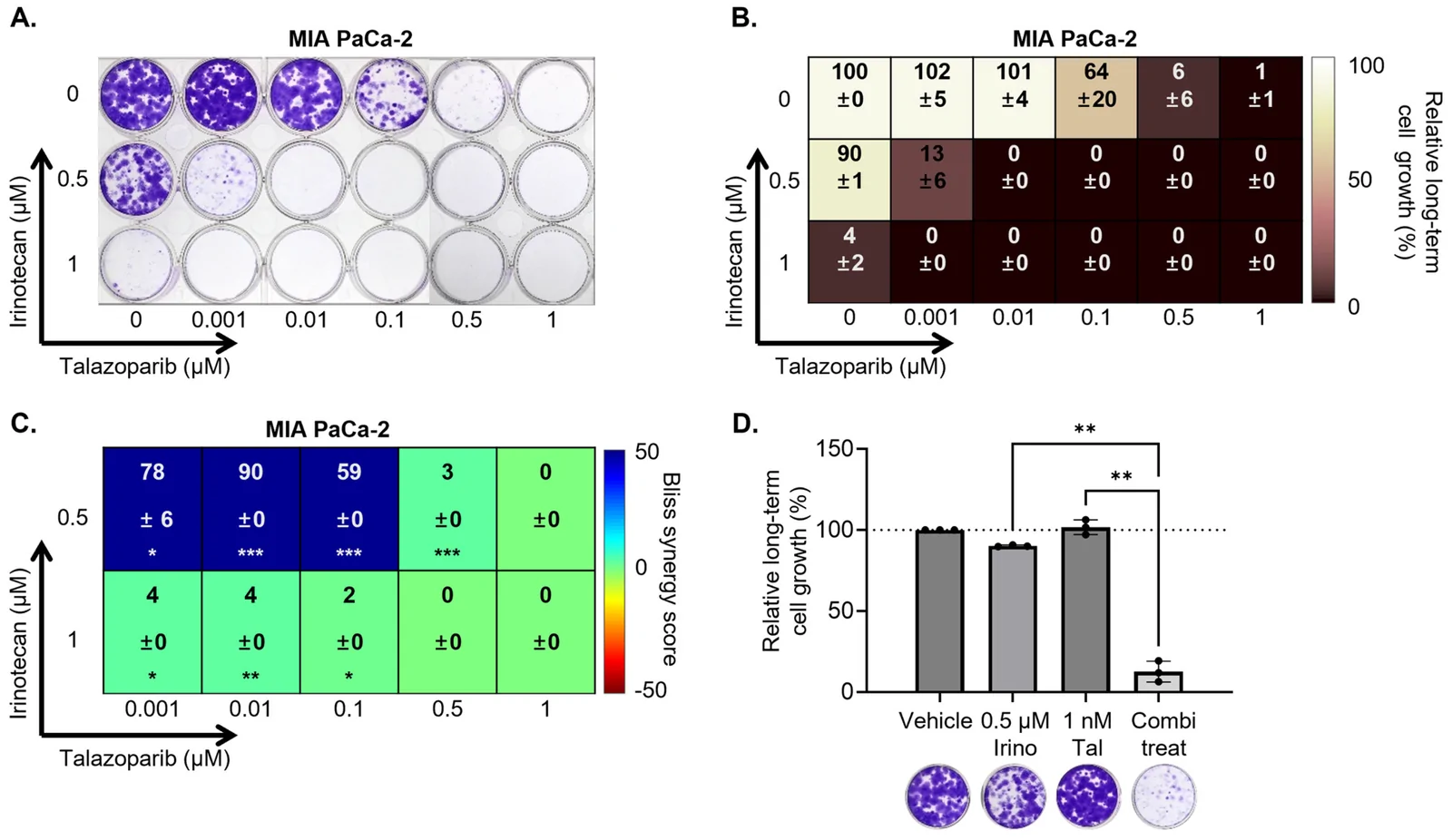
Long-term dose-matrix analysis of irinotecan and talazoparib in MIA PaCa-2 cells. (**A**) Representative crystal violet staining from one of three independent experiments in which MIA PaCa-2 cells were treated with the indicated concentrations of irinotecan and talazoparib for 10 days. Images from separate 12-well plates within the same experiment were assembled to display the complete concentration matrix. (**B**) Relative long-term cell growth quantified from crystal violet staining and normalized to vehicle-treated controls. Data are presented as mean ± SD from three independent experiments. (**C**) Bliss synergy scores calculated from the dose–response matrices. Data are presented as mean ± SD from three independent experiments. The color scale was limited to −50 to 50 for visualization; values outside this range are displayed at the corresponding scale limit, while the numerical values indicate the actual Bliss synergy scores. Statistical significance of Bliss synergy scores was assessed using a one-sample *t*-test against zero. * *p* < 0.05; ** *p* < 0.01; *** *p* < 0.001. (**D**) Relative long-term cell growth and representative crystal violet-stained wells under the indicated treatment conditions. Data are presented as mean ± SD from three independent experiments. Each dot represents one independent experiment. Statistical significance was determined by repeated-measures one-way ANOVA with Geisser–Greenhouse correction, followed by Šídák’s multiple comparisons test for selected pairwise comparisons. Irino, Irinotecan; Tal, Talazoparib; Combi, Combination.

**Figure 3:**
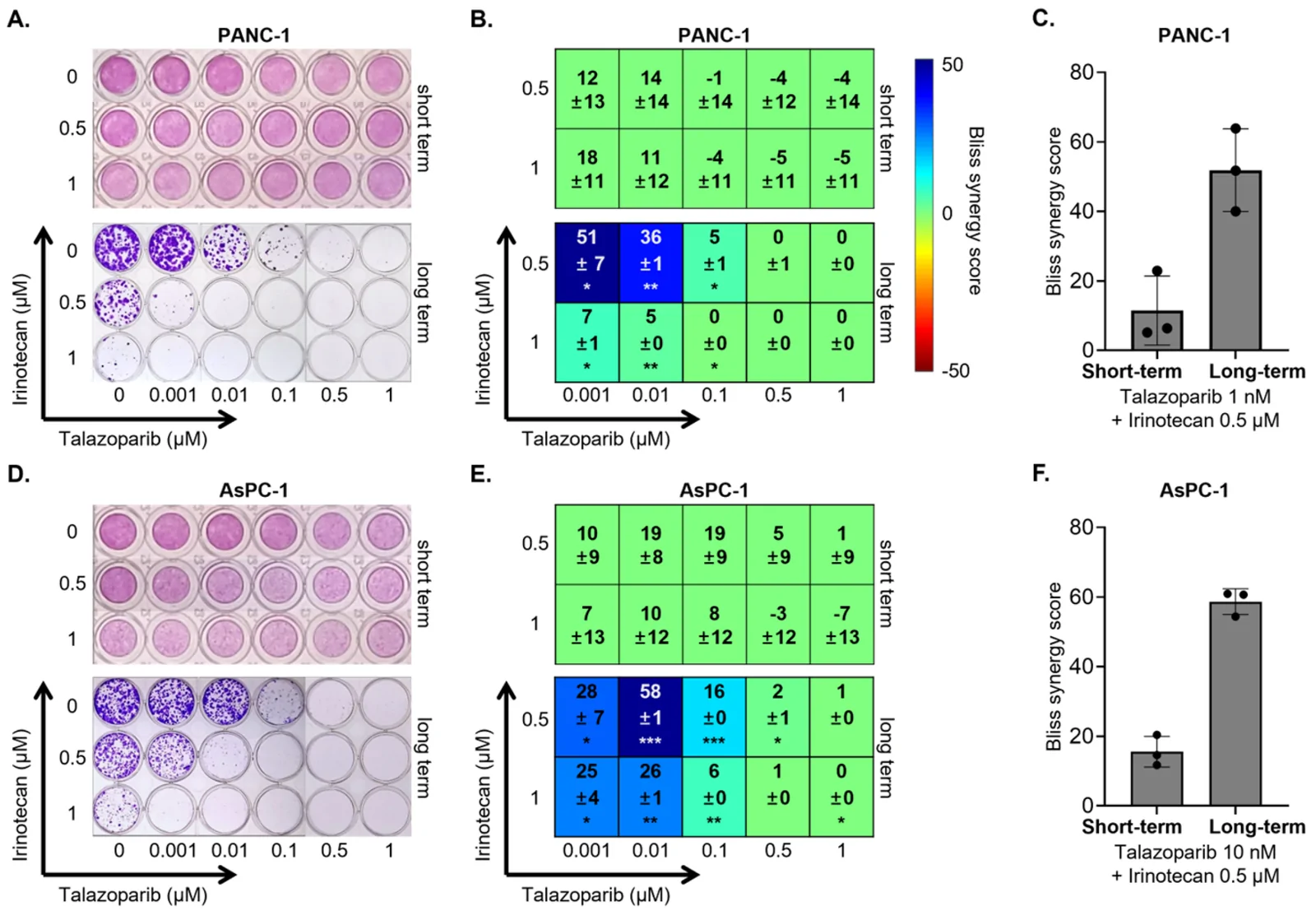
Comparison of short- and long-term responses to irinotecan plus talazoparib in PANC-1 and AsPC-1 cells. (**A**,**D**) Representative images of PANC-1 (**A**) and AsPC-1 (**D**) cells treated with concentration matrices of irinotecan and talazoparib in the 72 h SRB assay (top) and 10-day clonogenic assay (bottom). (**B**,**E**) Corresponding Bliss synergy score matrices for PANC-1 (**B**) and AsPC-1 (**E**) cells. Upper and lower matrices correspond to the short- and long-term assays, respectively. Values indicate mean ± SD from three independent experiments. * *p* < 0.05, ** *p* < 0.01, *** *p* < 0.001. (**C**,**F**) Bliss synergy scores at the indicated matched concentration pairs in the short- and long-term assays for PANC-1 (**C**) and AsPC-1 (**F**) cells. Each point represents one independent experiment.

**Figure 4:**
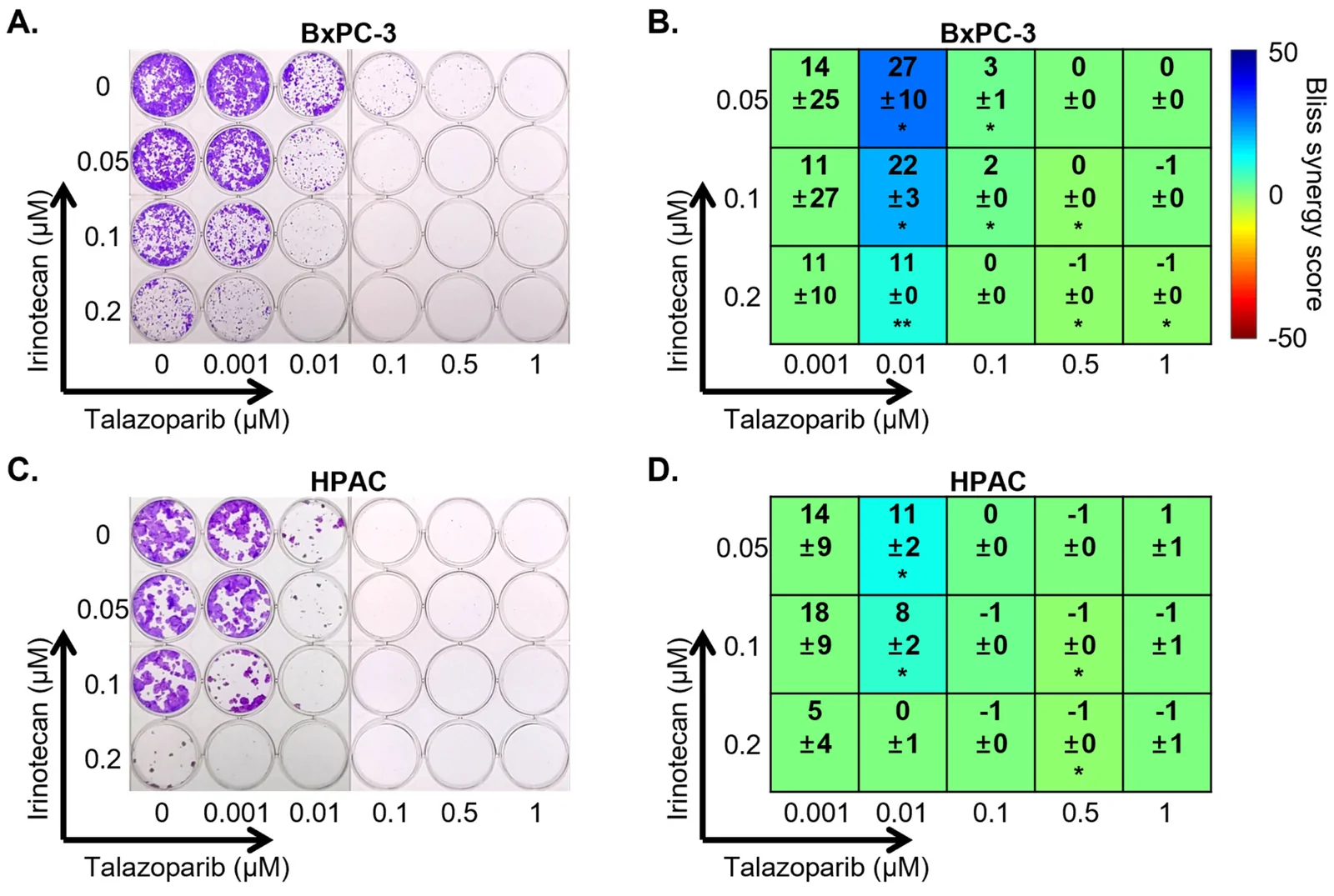
Long-term responses to irinotecan plus talazoparib in additional BRCA1/2-wild-type PDAC cell lines. (**A**,**C**) Representative colony-formation images of BxPC-3 (**A**) and HPAC (**C**) cells treated with concentration matrices of irinotecan and talazoparib for 10 days. (**B**,**D**) Corresponding Bliss synergy score matrices for BxPC-3 (**B**) and HPAC (**D**) cells. Values indicate mean Bliss synergy scores ± SD from three independent experiments. Statistical significance of Bliss synergy scores was assessed using a one-sample *t*-test against zero. * *p* < 0.05, ** *p* < 0.01.

**Figure 5:**
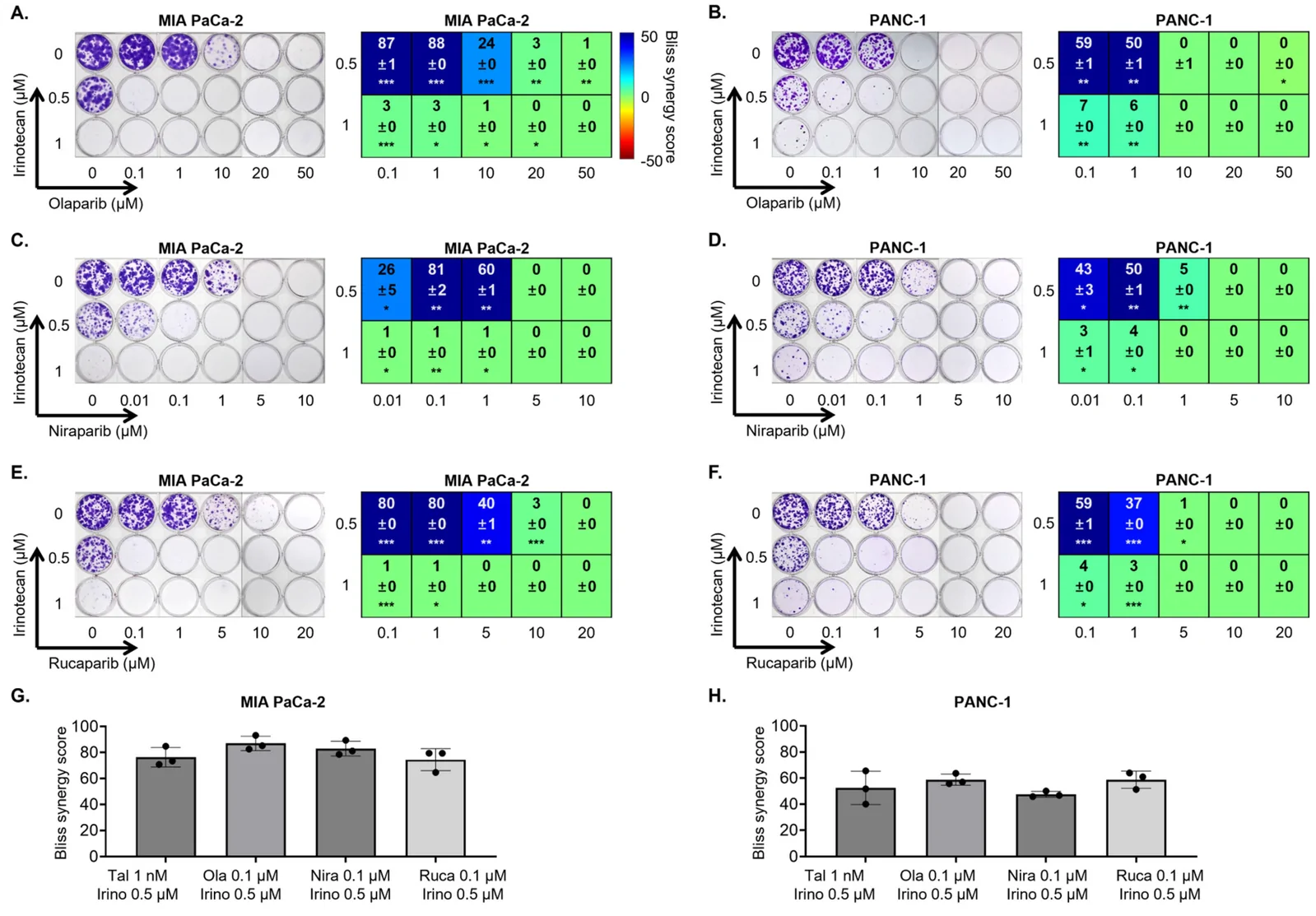
Multiple PARP inhibitors show long-term synergy with irinotecan in MIA PaCa-2 and PANC-1 cells. (**A**–**F**) Representative colony-formation images and corresponding Bliss synergy score matrices for irinotecan combined with olaparib (**A**,**B**), niraparib (**C**,**D**), or rucaparib (**E**,**F**) in MIA PaCa-2 (**A**,**C**,**E**) and PANC-1 (**B**,**D**,**F**) cells following 10 days of treatment. Values indicate mean Bliss synergy scores ± SD from three independent experiments. Statistical significance of Bliss synergy scores was assessed using a one-sample *t*-test against zero. * *p* < 0.05, ** *p* < 0.01, *** *p* < 0.001. (**G**,**H**) Bliss synergy scores for irinotecan paired with talazoparib, olaparib, niraparib, or rucaparib in MIA PaCa-2 (**G**) and PANC-1 (**H**) cells. For each PARP inhibitor, a representative concentration pair was selected from the dose–response matrix, and scores from the three independent experiments at this concentration pair are shown. Talazoparib data are included for comparison with the three additional PARP inhibitors. Each point represents one independent experiment, and data are presented as mean ± SD. Tal, talazoparib; Irino, irinotecan; Ola, olaparib; Nira, niraparib; Ruca, rucaparib.

**Figure 6:**
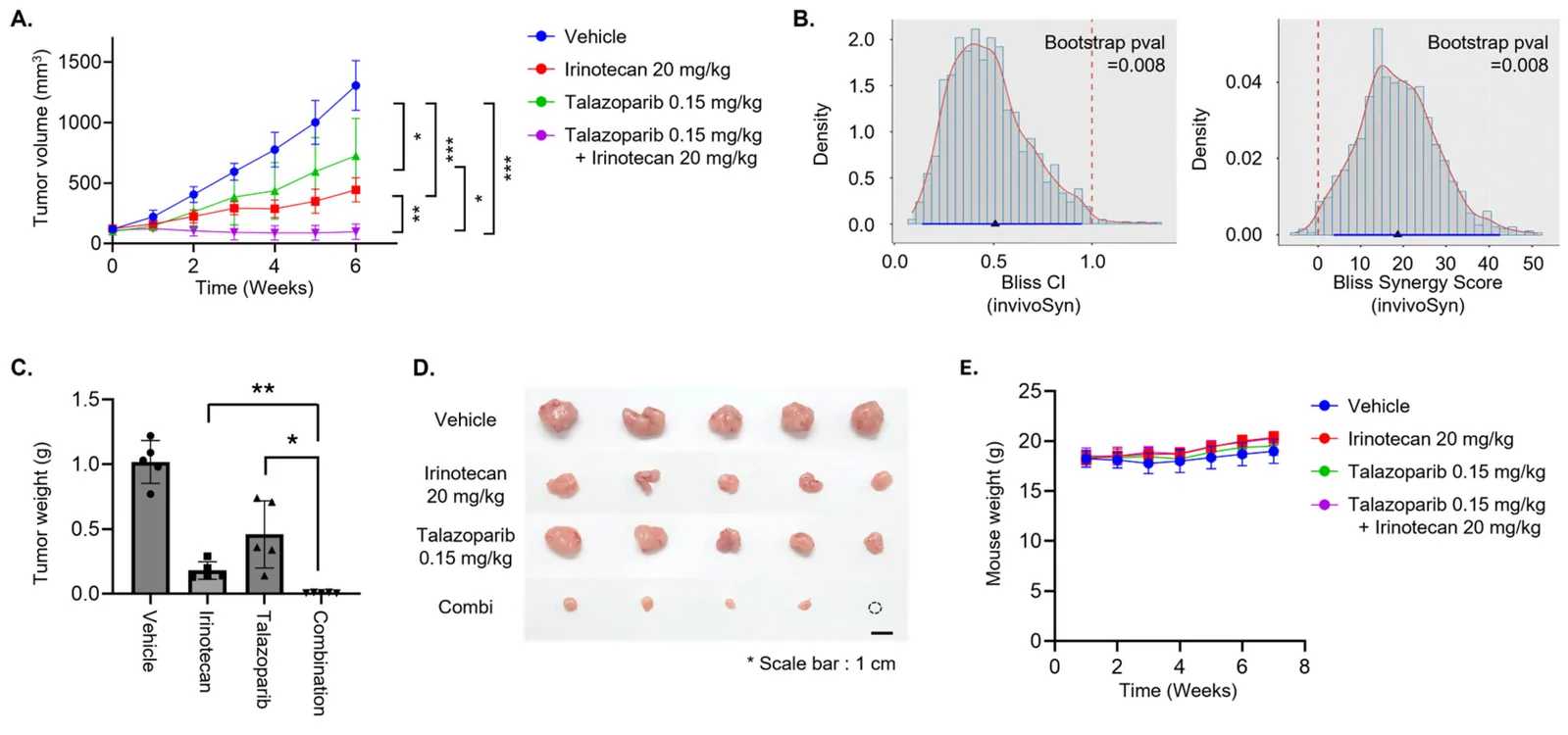
Talazoparib enhances the antitumor activity of irinotecan in an MIA PaCa-2 subcutaneous xenograft model. (**A**) Tumor-growth curves of MIA PaCa-2 subcutaneous xenografts treated with vehicle, irinotecan (20 mg/kg, intraperitoneally, once weekly), talazoparib (0.15 mg/kg, oral gavage, five times per week), or the combination for 6 weeks. Data are presented as mean ± SD; *n* = 5 mice per group. Statistical comparisons shown on the graph were based on tumor volumes at the final measurement time point and were performed using Welch’s one-way ANOVA followed by Dunnett’s T3 multiple-comparisons test. * *p* < 0.05, ** *p* < 0.01, *** *p* < 0.001. (**B**) Bootstrap distributions of the combination index (CI) and synergy score (SS) for irinotecan plus talazoparib calculated using invivoSyn based on the Bliss independence model. Histograms show the distributions of 1000 treatment-stratified bootstrap estimates, triangles indicate point estimates, horizontal lines indicate 95% BCa confidence intervals, and dashed lines indicate the additivity reference values of 1 for CI and 0 for SS. Bootstrap *p* values are shown in the plots. (**C**) Endpoint tumor weights for each treatment group. Each dot represents one mouse; data are presented as mean ± SD. Statistical significance was assessed using Welch’s unpaired *t*-tests for prespecified comparisons between the combination group and each single-agent group, with Holm–Šídák correction for multiple comparisons. (**D**) Gross images of tumors excised at the experimental endpoint. The black dashed circle indicates a mouse in which no grossly detectable tumor was observed at endpoint. Scale bar, 1 cm. (**E**) Body weight during the treatment period. Data are presented as mean ± SD; *n* = 5 mice per group.

**Figure 7:**
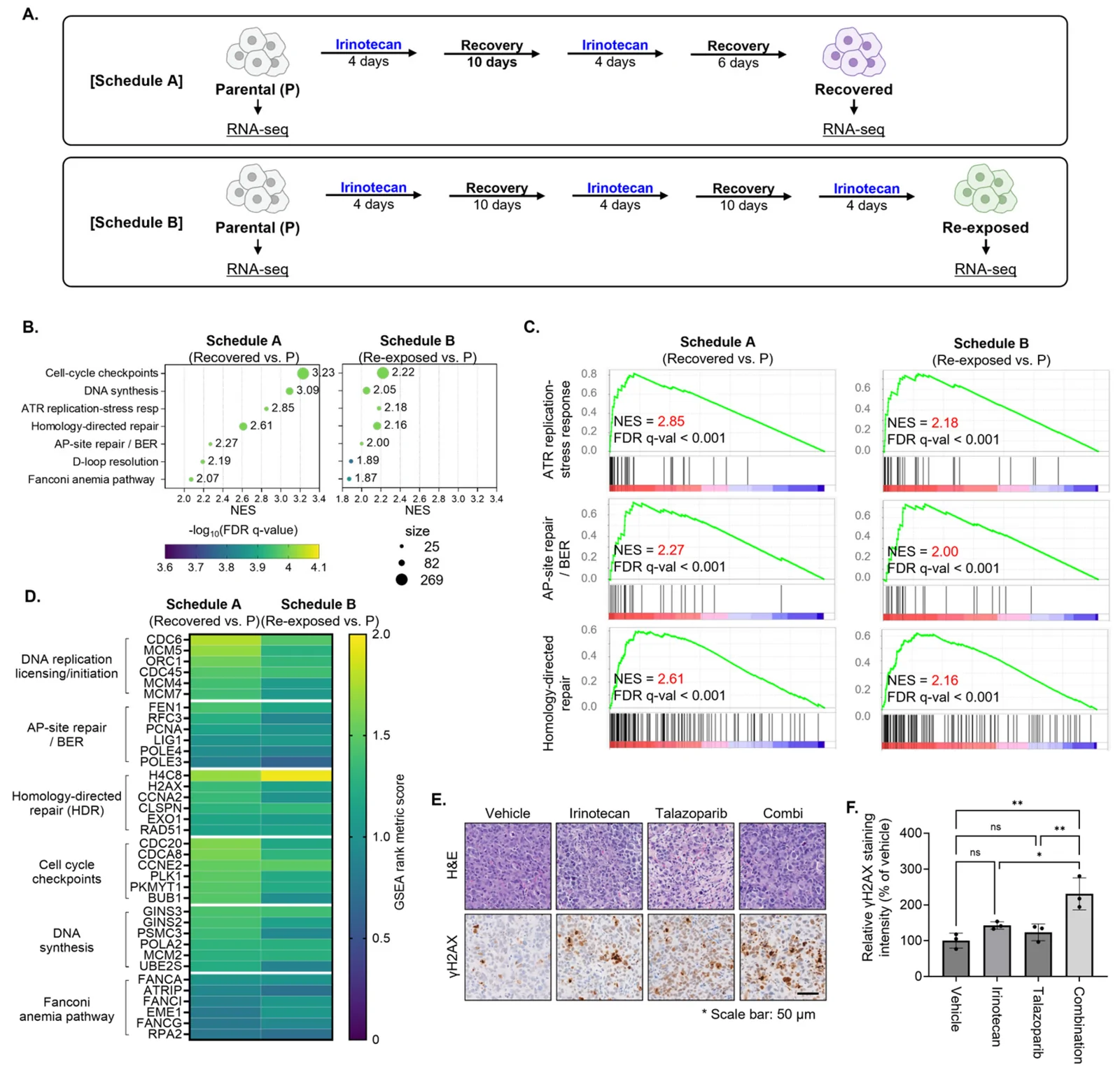
Irinotecan-conditioned cell populations show enrichment of replication-stress and DNA-repair programs, with increased γH2AX staining observed following irinotecan–talazoparib treatment in vivo. (**A**) Schematic representation of two irinotecan-conditioning schedules used for RNA-seq analysis in MIA PaCa-2 cells. In Schedule A, cells underwent two 4-day irinotecan treatments separated by a 10-day recovery period and were collected after a final 6-day recovery period (Recovered). In Schedule B, cells were treated with irinotecan for 4 days, allowed to recover for 10 days, treated again for 4 days, allowed to recover for another 10 days, and then re-exposed to irinotecan for 4 days before collection (Re-exposed). A single untreated parental sample (P) was used as the common reference for both schedules. (**B**) Gene set enrichment analysis (GSEA) of selected pathways related to cell-cycle regulation, DNA synthesis, replication stress, and DNA repair in Schedule A (Recovered vs. P) and Schedule B (Re-exposed vs. P). Dot position indicates the normalized enrichment score (NES), dot color indicates −log_10_(false discovery rate [FDR] q-value), and dot size represents gene-set size. (**C**) Representative GSEA enrichment plots for the ataxia telangiectasia and Rad3-related (ATR) replication-stress response, apurinic/apyrimidinic (AP)-site repair/base-excision repair (BER), and homology-directed repair (HDR) in Schedule A (Recovered vs. P) and Schedule B (Re-exposed vs. P). (**D**) Heatmap of GSEA rank metric scores for representative genes from the leading-edge subsets of pathways associated with DNA replication initiation, AP-site repair/repair synthesis, homology-directed repair-associated chromatin responses, cell-cycle checkpoints, DNA synthesis, and the Fanconi anemia pathway in Schedule A and Schedule B. Colors indicate the GSEA rank metric scores according to the color scale shown. (**E**) Representative hematoxylin and eosin (H&E) staining and γH2AX immunohistochemistry (IHC) of xenograft tumor tissues from vehicle, irinotecan, talazoparib, and combination-treated groups. Scale bar, 50 μm. (**F**) Quantification of γH2AX staining normalized to the vehicle group and expressed as relative γH2AX staining intensity. Data are presented as mean ± SD; each dot represents an individual tumor (*n* = 3 per group). * *p* < 0.05, ** *p* < 0.01.

## Data Availability

Data is contained within the article and the Supplementary Material.

## Supplementary Materials

The following supporting information can be downloaded at: https://www.mdpi.com/article/10.3390/ph19101538/s1, Figure S1: Relative growth responses and Bliss synergy scores for irinotecan–talazoparib combination treatment in PDAC cell lines; Figure S2: Long-term growth responses to irinotecan combined with additional PARP inhibitors in MIA PaCa-2 and PANC-1 cells; Table S1: List of 3524 compounds included in the drug screening library.

## Abbreviations

The following abbreviations are used in this manuscript:

ANOVA	analysis of variance
AP site	apurinic/apyrimidinic site
ATR	ataxia telangiectasia and Rad3-related
AUC	area under the curve
BCa	bias-corrected and accelerated
BER	base excision repair
CI	combination index
DAB	3,3′-diaminobenzidine
DMSO	dimethyl sulfoxide
eGR	exponential growth rate
FDA	U.S. Food and Drug Administration
FDR	false discovery rate
GSEA	gene set enrichment analysis
H&E	hematoxylin and eosin
HDR	homology-directed repair
IHC	immunohistochemistry
MSigDB	Molecular Signatures Database
NES	normalized enrichment score
PARP	poly(ADP-ribose) polymerase
PBS	phosphate-buffered saline
PDAC	pancreatic ductal adenocarcinoma
RNA-seq	RNA sequencing
SD	standard deviation
SRB	sulforhodamine B
SS	synergy score
T0	time zero
TPM	transcripts per million
γH2AX	phosphorylated histone H2AX at Ser139
