# Supplementary material for: Long-Term Drug Screening Identifies PARP Inhibitors as Irinotecan Combination Partners in BRCA1/2-Wild-Type Pancreatic Ductal Adenocarcinoma
Source: Pharmaceuticals (Basel). 2026 Sep 28;19(10):1538. doi: 10.3390/ph19101538 (PMC13708644; doi:10.3390/ph19101538)
Supplement: Supplementary file 1 [file pharmaceuticals-19-01538-s001.zip › pharmaceuticals-4570477-supplementary/Supplementary Information_pharmaceuticals_PARP inhibitor.pdf]

Supplementary Information

Long-Term Drug Screening Identifies PARP Inhibitors as Irinotecan Combination Partners in BRCA1/2-Wild-Type Pancreatic Ductal Adenocarcinoma

List of Supplementary Information

Supplementary Figure S1. Relative growth responses and Bliss synergy scores for irinotecan–talazoparib combination treatment in PDAC cell lines.

Supplementary Figure S2. Long-term growth responses to irinotecan combined with additional PARP inhibitors in MIA PaCa-2 and PANC-1 cells.

Supplementary Table S1. List of 3,524 compounds included in the drug screening library

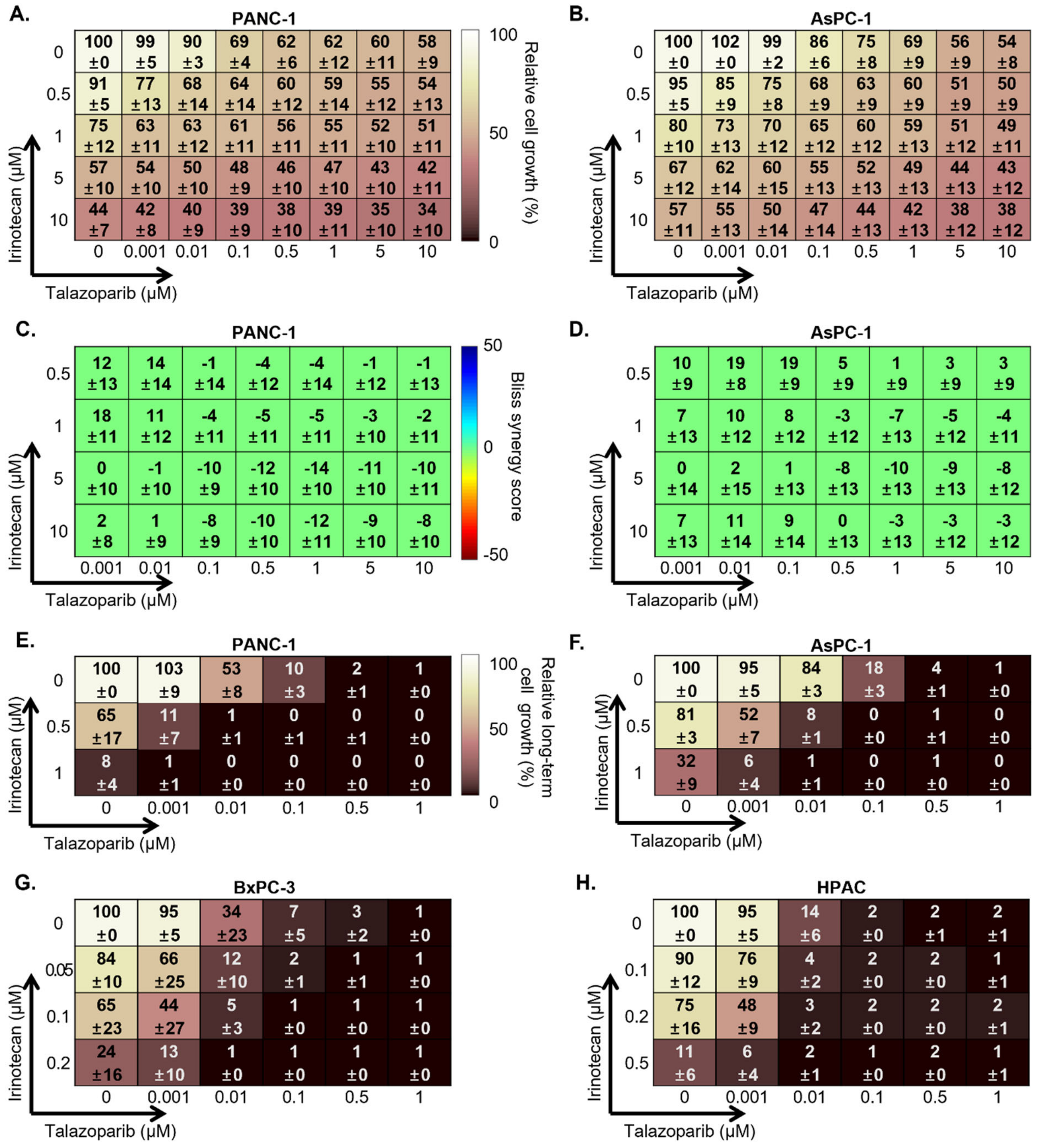

Supplementary Figure S1. Relative growth responses and Bliss synergy scores for irinotecan–talazoparib combination treatment in PDAC cell lines.

(A,B) Relative cell growth matrices of PANC-1 (A) and AsPC-1 (B) cells following 72-h treatment with concentration matrices of irinotecan and talazoparib. Relative cell growth was determined by SRB assay, expressed relative to the vehicle-treated control, and presented as mean ± SD from three independent experiments.

(C,D) Bliss synergy score matrices corresponding to the 72-h irinotecan–talazoparib combination assays in PANC-1 (C) and AsPC-1 (D) cells. Values indicate mean Bliss synergy scores ± SD from three independent experiments.

(E–H) Relative long-term cell growth matrices of PANC-1 (E), AsPC-1 (F), BxPC-3 (G) and

HPAC (H) cells following 10-day treatment with concentration matrices of irinotecan and talazoparib. Relative long-term cell growth was quantified by crystal violet staining, normalized to the corresponding vehicle-treated control, and presented as mean ± SD from three independent experiments.

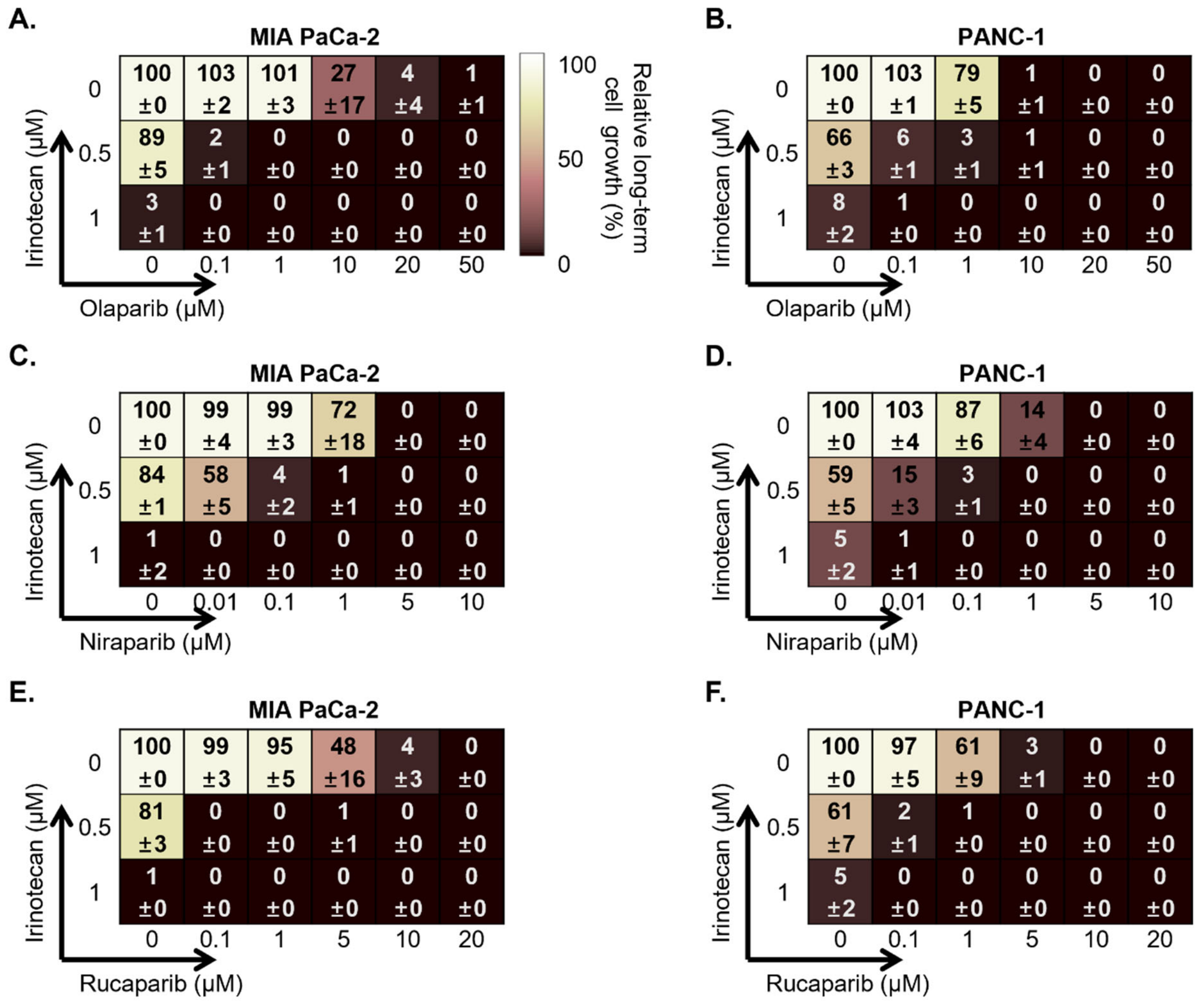

Supplementary Figure S2. Long-term growth responses to irinotecan combined with additional PARP inhibitors in MIA PaCa-2 and PANC-1 cells.

(A–F) Relative long-term cell growth matrices of MIA PaCa-2 (A,C,E) and PANC-1 (B,D,F) cells following 10-day treatment with concentration matrices of irinotecan and olaparib (A,B), niraparib (C,D), or rucaparib (E,F). Values were normalized to the corresponding vehicle-treated control and are presented as mean ± SD from three independent experiments.

Supplementary Table S1. List of 3,524 compounds included in the drug screening library

Table S1 is provided as a separate Excel file.
